# Obstetric Care and Method of Delivery in Mexico: Results from the 2012 National Health and Nutrition Survey

**DOI:** 10.1371/journal.pone.0104166

**Published:** 2014-08-07

**Authors:** Ileana Heredia-Pi, Edson E. Servan-Mori, Veronika J. Wirtz, Leticia Avila-Burgos, Rafael Lozano

**Affiliations:** 1 National Institute of Public Health, Cuernavaca, Morelos, Mexico; 2 Center for Global Health and Development, Boston University, Boston, Massachusetts, United States of America; 3 Institute for Health Metrics and Evaluation, Seattle, Washington, United States of America; University of Florida, United States of America

## Abstract

**Objective:**

To identify the current clinical, socio-demographic and obstetric factors associated with the various types of delivery strategies in Mexico.

**Materials and Methods:**

This is a cross-sectional study based on the 2012 National Health and Nutrition Survey (ENSANUT) of 6,736 women aged 12 to 49 years. Delivery types discussed in this paper include vaginal delivery, emergency cesarean section and planned cesarean section. Using bivariate analyses, sub-population group differences were identified. Logistic regression models were applied, including both binary and multinomial outcome variables from the survey. The logistic regression results identify those covariates associated with the type of delivery.

**Results:**

53.1% of institutional births in the period 2006 through 2012 were vaginal deliveries, 46.9% were either a planned or emergency cesarean sections. The highest rates of this procedure were among women who reported a complication during delivery (OR: 4.21; 95%CI: 3.66–4.84), between the ages of 35 and 49 at the time of their last child birth (OR: 2.54; 95%CI: 2.02–3.20) and women receiving care through private healthcare providers during delivery (OR: 2.36; 95%CI: 1.84–3.03).

**Conclusions:**

The existence of different socio-demographic and obstetric profiles among women who receive care for vaginal or cesarean delivery, are supported by the findings of the present study. The frequency of vaginal delivery is higher in indigenous women, when the care provider is public and, in women with two or more children at time of the most recent child birth. Planned cesarean deliveries are positively associated with years of schooling, a higher socioeconomic level, and higher age. The occurrence of emergency cesarean sections is elevated in women with a diagnosis of a health issue during pregnancy or delivery, and it is reduced in highly marginalized settings.

## Introduction

There are many factors that contribute to the decision to deliver either vaginally or through a cesarean section in a health facility. According to the conceptual framework proposed by Freitas [1], these levels include the individual socio-demographic and cultural characteristics of the woman, her obstetric and prenatal care history, her preferences, as well as the circumstances at the time of admission to a facility and throughout the delivery process. Factors within the facility are staff member training and general facility characteristics [1], [2].

In recent decades, the number of deliveries within health care facilities in Mexico has increased, from 78.7% in 1990 to 94.1% and 95.1% in 2010 and 2012, respectively [3], [4]. During the same time period, the percentage of pregnancies ending in a cesarean section delivery has risen markedly [5]. This trend is present in both private and public health facilities across the country. Mexico has one of the highest cesarean delivery rates internationally [6]–[8]. According to the OECD, in 2011 Mexico was the member country with the highest rate of cesarean sections (49 cesarean sections/100 deliveries) [5], [8]. UNICEF estimations for 2012 confirmed this, “from 2006 to 2010, Mexico had the second highest rate of cesarean sections within the Americas, with 43 cesarean sections per 100 deliveries. This rate was only surpassed by Brazil with a rate of 50/100 deliveries” [9].

The trend of increasing reliance on cesarean sections is not exclusive to Mexico, rather, it is a global public health issue especially relevant to low and middle income countries with low health budgets compared to high income countries [10]–[14]. Concern about the overreliance on cesarean section is based on, first, the association between the procedure and both maternal and fetal medical complications and second, the higher expenditure which does not result in better health outcomes [15]. Cesarean sections are associated with increased neonatal mortality (OR: 1.7; 95%CI: 1.3–2.2 for intra-delivery cesarean section and OR: 1.9; 95%CI: 1.5–2.6 for elective cesarean section, both compared to vaginal delivery), respiratory complications and neurological deficits in newborn infants. Beyond the health complications for the infant, cesarean sections are also associated with increased maternal morbidity and mortality as well as posing a risk for complications in future pregnancies (OR: 2.0; 95%CI: 1.6–2.5 for intra-delivery cesarean section and OR: 2.3; 95%CI: 1.7–3.1 for elective cesarean section, both compared to vaginal delivery) [15]. The financial burden of cesarean sections on the health system is high, with greater utilization of medical personnel resources related to the surgical procedure compared to a vaginal delivery [16].

Previous studies have documented factors associated with vaginal delivery or cesarean section. On the demand side, individual characteristics of the women (demographic, socioeconomic status, type of medical insurance, obstetric history, etc.) are very important, whereas on the supply side, associated factors are type of health institution (public or private), availability of new technologies at the medical units (utilization of the cardiac fetal electronic monitoring, blood transfusions, antibiotics, etc.), clinical staff available at the health unit (obstetricians, anesthetists, etc.), and others [11]–[18].

Health facility statistics have historically been the primary source of information used to study the prevalence of different delivery methods. However, the mothers’ socio-demographic characteristics and the obstetric factors that influence the delivery strategy have not yet been thoroughly investigated through population surveys in Mexico [13]–[18]. This is a cross-sectional study based on the 2012 National Health and Nutrition Survey (ENSANUT) with the objective to identify the current clinical, socio-demographic and obstetric factors associated with the various types of delivery strategies in Mexico.

## Materials and Methods

A cross-sectional study was conducted based on the data collected through the 2012 National Health and Nutrition Survey (ENSANUT), a probabilistic survey that is representative at the national and state level, as well as by urban/rural stratum. The data for the analysis were requested and obtained from the surveys public repository hosted at the National Institute of Public Health (NIPH) (See webpage at: http://ensanut.insp.mx/). The Institutional Board (Ethics Committee) at the NIPH in Mexico reviewed and approved the survey. All interviewees provided informed consent prior to participating.

This survey was administered to 194, 758 Mexicans, allowing the analysis of their health status and health protection coverage, and the health system’s performance in Mexico [19]. In particular, the sections applied to teenagers and adults were analyzed. Information on reproductive health was collected from a sample of 58,391 women aged 12–49 years, from which a random sample was selected (26,261) who responded to an antenatal care questionnaire. Out of these 7,884 reported a live childbirth occurring in the period 2006 through 2012 and 6,736 were included in the study analysis. Cases with missing data were excluded (237 teenagers and 911 adults) as well as women reporting deliveries outside health institutions. No difference in the study variables was found between those individuals included in the data analysis and those who were excluded due to missing data. In some cases not all of the factors examined in this analysis were available for all women.

### Outcome variable: Type of delivery

The type of delivery was defined based on the individual self-report by the surveyed women regarding their delivery experience at the last obstetric event. The ENSANUT questionnaire asked this information using the following question “Was your last delivery…?” to which the women could reply normal (equivalent to vaginal delivery), emergency cesarean section, or planned cesarean section.

#### Associated factors

Factors identified in the literature as associated with the likelihood of receiving medical care and/or utilizing health services during the prenatal or delivery stages in low- and middle-income countries were included in the study [20]–[26]. These factors were divided into three groups:

- General socio-demographic factors: These include individual, household, and neighborhood characteristics. The factors considered were: years of schooling (zero, 1–6, 7–9, ≥10); marital status; health insurance (Social Security-SS, Seguro Popular (SP), more than one, and none); residence in an indigenous household [27], annual expenses per resident (Exp-pc) in quintiles, indicator for being a part of a beneficiary household of the Oportunidades program (formerly PROGRESA) and the population size of the neighborhood as defined by metropolitan-urban-rural status. Rural neighborhoods were defined as locations with less than 2500 inhabitants, urban areas were those with 2500 to 100,000 inhabitants, and metropolitan locations were areas with greater than 100,000 residents. Degree of the municipality deprivation or marginalization (in percentage, based on access to basic infrastructure services, housing conditions, education attainment, and wage earnings, at locality level) was also considered in this analysis [28].
- Respondent characteristics (sociodemographic factors and obstetric history) at time of the most recent child birth: Covariates in this category include the age of the woman (12–19, 20–34, 35–49 yrs.), the number of children (0, 1, and ≥2) at time of the most recent child birth, having had at least one stillborn child or a child who died before the first year of life, and a history of abortion.
- Characteristics related to prenatal and delivery care: Attending at least four antenatal care visits (ANC), having the first ANC visit within the first trimester of pregnancy, the frequent care provider as SS (including the Mexican Institute of Social Security or IMSS, the Institute of Social Security and Services for the State Workers or ISSSTE, and other social security institutions), the Secretariat of Health, and Private; a high blood pressure, vaginal bleeding, anemia, threat of miscarriage, preeclampsia or eclampsia, gestational diabetes, or infections during pregnancy. Covariates specific at the time of the childbirth include: the place of care, and having reported a complication (preeclampsia or eclampsia, hemorrhage, miscarriage, threat of miscarriage, obstructed delivery, wrong position of the fetus, premature childbirth, or some complication due to a previous disease).

### Statistical analysis

Factors associated with the type of delivery were identified based on bivariate analyses (χ^2^ for categorical variables and t-student tests for continuous variables), to determine the main differences between the respondent’s that received each type of delivery method.

Logistic regression with both a binary and a multinomial outcome variable were used to identify the factors that are significantly associated with the delivery method a mother receives in a hospital setting [29], [30]. In the logistic multivariate model, the response variable was 1 if the delivery was a cesarean section and 0 if the delivery was a vaginal delivery. It included individual socio-demographic and household characteristics, as well as covariates related to the neighborhood of residence (urban, rural, and metropolitan); characteristics of the respondents that related to her pregnancy history and the prenatal period and the pregnancy and delivery care indicators. For the multinomial regression, the response variable was defined as zero for vaginal delivery, as one for emergency cesarean section, and as two for planned cesarean section, the first being the reference category, all of the covariates included in binary model were included in the multinomial regression. Furthermore, all the estimated models included the geographic region where the respondent resides (northwest, northeast, central-north, east, west, central-south, southwest, southeast). Stata SE v13.1 software was used for the analysis in this study [31].

## Results

The socio-demographic profile of the study population, the obstetric background at the time of the last childbirth and health care indicators, according to the type of delivery, are shown in Tables 1–3. 53.1% of the women mentioned having given birth via vaginal delivery, and 46.9%, via cesarean section. 27.9% of the women affiliated to SS (95%CI: 24.5–31.5) declared having received care for a planned cesarean section, while only 14.6% (95%CI: 13.0–16.4) of the women affiliated to the SP received such care; the percentage in this group was statistically lower than those observed in women with other types of health insurance. Indigenous women and those belonging to the first quintile of the Exp-pc reported the highest percentage of vaginal deliveries (around 62%). This percentage is statistically higher than the one observed for strata 4 and 5 and among non-indigenous women.

**Table 1:** Sociodemographic profile of the study population, by type of delivery.

	Vaginal delivery	Type of cesarean section		p-value*
		Emergency	Planned	
**Sample**	3,777	1,702	1,257	
** Weighted sample**	5,095,628	2,515,187	1,985,472	
** **%	53.1	26.2	20.7	
National**	53.1 [51.3,54.9]	26.2 [24.6,27.9]	20.7 [19.1,22.4]	----
Characteristics of the women				
Schooling (yrs.)				
Zero	57.6 [45.3,69.0]	30.1 [21.1,40.9]	12.3 [7.12,20.5]	0.00
1–6	59.9 [56.2,63.5]	26.0 [22.9,29.4]	14.1 [11.8,16.8]	
7–9	57.9 [55.1,60.7]	25.1 [22.7,27.7]	17.0 [14.8,19.4]	
≥10	43.9 [40.8,47.0]	27.1 [24.3,30.1]	29.1 [26.1,32.2]	
Health insurance				
Social Security	45.6 [42.2,49.0]	26.6 [23.7,29.6]	27.9 [24.5,31.5]	0.00
Seguro Popular (SP)	59.1 [56.8,61.4]	26.3 [24.3,28.4]	14.6 [13.0,16.4]	
Mixed	52.6 [41.2,63.7]	30.2 [21.7,40.5]	17.2 [10.7,26.5]	
None	51.0 [46.4,55.6]	25.2 [21.3,29.6]	23.8 [20.3,27.8]	
Characteristics of the household of residence				
Indigenous				
No	52.3 [50.4,54.2]	26.2 [24.5,27.9]	21.5 [19.8,23.3]	0.00
Yes	61.6 [55.5,67.3]	26.6 [21.6,32.2]	11.8 [8.73,15.8]	
Expenditure quintiles per resident/yr.				
I	62.7 [58.9,66.4]	24.3 [20.9,28.0]	13.0 [10.7,15.8]	0.00
II	55.3 [51.2,59.4]	26.1 [24.4,30.1]	18.6 [15.4,22.2]	
III	57.8 [53.7,61.8]	24.4 [21.1,27.9]	17.9 [14.6,21.7]	
IV	50.5 [46.0,55.1]	28.6 [25.0,32.5]	20.9 [17.6,24.6]	
V	39.2 [35.3,43.2]	27.7 [24.2,31.6]	33.1 [28.8,37.6]	
Oportunidades beneficiary				
No	50.8 [48.7,53.0]	27.0 [25.0,29.0]	22.2 [20.3,24.3]	0.00
Yes	61.5 [58.0,64.9]	23.5 [20.6,26.6]	15.1 [12.6,18.0]	
Characteristics of the place of residence				
Rural (<2,500 inhab.)	60.5 [57.4,63.5]	24.6 [21.9,27.5]	14.9 [12.5,17.6]	0.00
Urban (2,500–100,000 inhab.)	51.0 [47.9,54.1]	30.0 [26.9,33.2]	19.0 [16.7,21.6]	
Metropolitan (>100,000 inhab.)	51.1 [48.3,53.8]	25.5 [23.1,28.0]	23.5 [21.0,26.1]	
Low marginalization	50.9 [48.7,53.1]	27.0 [25.0,29.0]	22.2 [20.2,24.2]	
High marginalization	60.6 [57.7,63.5]	23.7 [21.2,26.4]	15.7 [13.3,18.4]	

Note: estimations based on the effect of the survey design.

*Refer to differences between vaginal delivery, and emergency and planned type of cesarean section.

**Refer to national level estimations.

**Table 2:** Sociodemographic and obstetric background at the time of the last childbirthφ.

	Vaginaldelivery	Type of cesarean section		p-value*
		Emergency	Planned	
**Sample**	3,777	1,702	1,257	
** Weighted sample**	5,095,628	2,515,187	1,985,472	
Age (yrs.)				
12–19	60.2 [55.9,64.4]	28.6 [24.9,32.5]	11.2 [8.72,14.4]	0.00
20–34	52.1 [49.9,54.4]	26.0 [24.0,28.1]	21.9 [19.9,24.0]	
35–49	45.3 [39.7,50.9]	22.8 [18.6,27.7]	31.9 [26.9,37.4]	
Number of children				
0	46.8 [43.4,50.3]	34.4 [31.3,37.7]	18.8 [15.7,22.3]	0.00
1	49.3 [45.9,52.7]	26.2 [23.2,29.4]	24.5 [21.8,27.5]	
≥2	62.7 [59.7,65.5]	18.5 [16.4,20.8]	18.9 [16.6,21.4]	
Child dead at childbirth or during the 1^st^ yr.				
No	53.4 [51.5,55.3]	26.0 [24.3,27.8]	20.5 [18.9,22.3]	0.30
Yes	47.1 [39.3,55.0]	29.5 [23.0,36.9]	23.4 [17.6,30.5]	
At least one abortion				
No	53.8 [51.7,55.9]	26.3 [24.5,28.2]	19.9 [18.1,21.8]	0.03
Yes	49.2 [45.0,53.4]	25.7 [22.1,29.6]	25.1 [21.6,29.1]	

Note: Estimations based on the effect of the survey design.

φ At time of the most recent child birth.

*Refer to differences between vaginal delivery, and emergency and planned type of cesarean section.

**Table 3:** Care indicators during the last pregnancy and delivery.

	Vaginal delivery	Type of cesarean section		p-value*
		Emergency	Planned	
**Sample**	3,777	1,702	1,257	
** Weighted sample**	5,095,628	2,515,187	1,985,472	
Prenatal				
At least four consultations				
No	62.5 [55.1,69.4]	28.5 [22.5,35.2]	9.03 [5.66,14.1]	0.00
Yes	52.4 [50.5,54.3]	26.0 [24.3,27.8]	21.6 [19.9,23.4]	
First consultation during the 1^st^ quarter				
No	59.6 [54.9,64.2]	27.3 [23.3,31.8]	13.1 [10.1,16.8]	0.00
Yes	51.9 [49.8,54.0]	26.0 [24.2,27.9]	22.1 [20.3,24.0]	
Frequent care provider				
Secretariat of Health	62.6 [60.2,64.9]	24.3 [22.3,26.5]	13.1 [11.6,14.9]	0.00
Social Security	51.4 [47.6,55.2]	29.7 [26.2,33.6]	18.9 [16.2,21.9]	
Private	36.0 [31.9,40.2]	26.0 [22.2,30.2]	38.1 [33.7,42.6]	
Diagnosis of some health problem				
No	57.2 [54.2,60.2]	21.3 [18.9,23.8]	21.5 [19.0,24.3]	0.00
Yes	50.3 [47.8,52.8]	29.6 [27.4,31.9]	20.1 [18.1,22.3]	
Moment of childbirth				
Place of care				
Secretariat of Health	63.4 [61.1,65.6]	23.6 [21.7,25.7]	13.0 [11.4,14.7]	0.00
Social Security	52.7 [49.1,56.3]	29.6 [26.2,33.2]	17.7 [15.3,20.4]	
Private	29.9 [26.1,34.0]	27.5 [23.2,32.3]	42.6 [38.0,47.3]	
Complication during childbirth				
No	59.4 [57.3,61.4]	18.1 [16.5,19.8]	22.6 [20.7,24.5]	0.00
Yes	28.5 [25.0,32.4]	58.1 [53.9,62.2]	13.4 [10.4,17.0]	

Note: estimations based on the effect of the survey design.

*Refer to differences between vaginal delivery, and emergency and planned type of cesarean section.

According to the obstetric history at the time of the last child birth, as the age of the women increases, there is a tendency for planned cesarean section over vaginal deliveries, with a statistically significant difference compared to younger women. On the other hand, as a respondent’s total number of children increases, vaginal delivery becomes more frequent and cesarean section less so. Within the women that received an emergency cesarean section, less woman receive the procedure as the number of children they have previously given birth to increases (Table 2).

Table 3 shows women who did not have at least 4 prenatal care visits, with 62.5% (95%CI: 55.1–69.4) of vaginal deliveries reported. In contrast, women who received ≥4 ANC visits had a lower frequency of vaginal delivery and a higher frequency of planned cesarean sections (with statistical significance). Delivery method differed by the type of health care facility the women used during prenatal care, with 64.1% of the women who received care from the private sector reporting a cesarean section (26.0% emergencies and 38.1% planned). Unlike, the lowest percentage of cesarean sections (37.4%) was reported from women receiving antenatal care from the Secretariat of Health (24.3% emergencies and 13.1% planned). According to the care provider, at time of the most recent child birth, 70.1% of the women who received care from the private sector reported cesarean sections (27.5% emergencies and 42.6% planned), followed by those who received care at SS units (47.6%; 29.6% emergencies and 17.7% planned). When a complication during birth occurred, higher rates of emergency cesarean sections were observed (58.1%) versus only 18.1% when no event of complication occurred.

**Figure 1:**
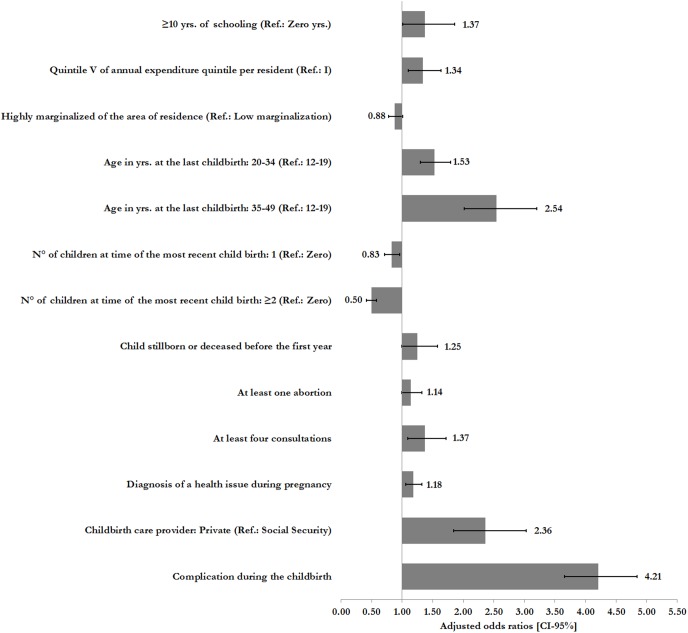
Sociodemographic and obstetric factors associated with cesarean section^‡^. Source: Mexican National Nutrition Survey, 2012. Note: ^‡^Adjusted logistic model. Estimates controlling for fixed effects by geographical region (Northwest, Northeast, Central-North, East, West, Central-South, Southwest, and Southeast). No statistically significant variables (p<0.10): 1–6 and 7–9 yrs. of schooling; Health Insurance; Indigenous (Ref.: Non-indigenous); Beneficiary of the Oportunidades program (Ref.: Non-beneficiary); Quantile of annual expenditure per resident: II–III; Urban and metropolitan area (Ref.: Rural); First prenatal consultation during the 1st trimester; Frequent prenatal care provider: Secretariat of Health and Private (Ref.: Social Security); Childbirth care provider: Secretariat of Health (Ref.: Social Security). Adjustment statistics: Akaike (AIC) = 8,159; Log likelihood = −4,042; Hosmer-Lemeshow χ2 = 12.8 (Prob> χ2 = 0.118).

The results of the multivariate estimated models are shown in Figure 1 and Table 4. When socio-demographic and obstetric factors associated with cesarean section were analyzed (Figure 1), results show that women who reported a complication during delivery had four times the risk of receiving a cesarean section than those who reported no complications (OR: 4.21; 95%CI: 3.66–4.84). The next highest odds of receiving a cesarean section was in women between the ages of 35 and 49 at the time of their most recent child birth (OR: 2.54; 95%CI: 2.02–3.20) and women receiving care through private healthcare providers during delivery (OR: 2.36; 95%CI: 1.84–3.03). The likelihood of receiving care in the form of a cesarean section is significantly lower in women who live in areas with high (compared to low) marginalization (OR: 0.88; 95%CI: 0.78–1.01) and in women who reported having one children (OR: 0.83; 95%CI: 0.71–0.96) or 2 or more children, at the time of their last birth, (OR: 0.50; 95%CI: 0.42–0.58).

**Table 4:** Sociodemographic and obstetric factors associated with the type of delivery^‡^.

	Type of delivery (Ref.: Vaginal delivery)			
	Emergency c-section		Planned c-section	
	Odds ratios [CI-95%] reported			
Characteristics of the mother				
Years of schooling (Ref.: Zero yrs.)				
1–6	1.05	[0.74,1.49]	1.37	[0.87,2.15]
7–9	1.02	[0.72,1.44]	1.67*	[1.07,2.59]
≥10	1.02	[0.71,1.47]	2.04**	[1.30,3.20]
Health insurance (Ref.: None)				
Social Security	1.17	[0.93,1.46]	1.05	[0.84,1.32]
Seguro Popular (SP)	1.18+	[0.97,1.44]	0.88	[0.71,1.08]
Mixed	1.42	[0.92,2.18]	0.61+	[0.37,1.03]
Characteristics of the household of residence				
Indigenous (Ref.: Non-indigenous)	0.93	[0.74,1.16]	0.74*	[0.56,0.99]
Beneficiary of the Oportunidades program (Ref.: Non-beneficiary)	0.99	[0.84,1.17]	0.82*	[0.68,1.00]
Annual expenditure quintile per resident (Ref.: I)				
II	1.02	[0.84,1.24]	1.02	[0.82,1.28]
III	0.98	[0.80,1.24]	0.92	[0.73,1.16]
IV	1.10	[0.88,1.36]	1.14	[0.90,1.45]
V	1.27*	[1.00,1.61]	1.42**	[1.11,1.83]
Characteristics of the area of residence				
Urban (2,500–100,000 inhab.) (Ref.: Rural)	1.11	[0.93,1.33]	1.11	[0.91,1.37]
Metropolitan (>100,000 inhab.) (Ref.: Rural)	1.01	[0.85,1.21]	1.06	[0.87,1.29]
Highly marginalized (Ref.: Low marginalization)	0.86+	[0.74,1.00]	0.93	[0.78,1.10]
History during the last pregnancy/child birthφ				
Age in yrs. at the last childbirth (Ref.: 12–19)				
20–34	1.42**	[1.18,1.70]	1.80**	[1.44,2.25]
35–49	2.13**	[1.62,2.80]	3.26**	[2.42,4.39]
No of children at time of the most recent child birth (Ref.: Zero)				
1	0.58**	[0.49,0.69]	1.35*	[1.11,1.64]
≥2	0.34**	[0.28,0.41]	0.85	[0.69,1.05]
Child stillborn or deceased before the first year	1.27+	[0.96,1.67]	1.23	[0.91,1.67]
At least one abortion	1.04	[0.87,1.24]	1.25*	[1.05,1.49]
At least four consultations	1.19	[0.91,1.55]	1.77**	[1.24,2.52]
First prenatal consultation during the 1^st^ trimester	0.94	[0.78,1.12]	1.21+	[0.98,1.51]
Frequent prenatal care provider (Ref.: Social Security)				
Secretariat of Health	0.92	[0.69,1.24]	0.83	[0.60,1.14]
Private	1.01	[0.75,1.36]	1.34*	[1.00,1.81]
Diagnosis of a health issue during pregnancy	1.32**	[1.15,1.51]	1.05	[0.91,1.21]
Childbirth care (Ref.: Social Security)				
Secretariat of Health	0.80	[0.60,1.07]	1.04	[0.77,1.42]
Private	1.83**	[1.35,2.47]	2.86**	[2.12,3.87]
Complication during the childbirth	6.93**	[5.96,8.05]	1.46**	[1.19,1.78]
Sample	6,736			
AIC	11,626			
Log likelihood	−5,739			
McFadden’s R^2^	0.14			
χ^2^	17.4			
Prob>χ^2^	0.36			

Note: ^‡^estimates controlling for fixed effects by geographical region (Northwest, Northeast, Central-North, East, West, Central-South, Southwest, Southeast).

φ At time of the most recent child birth. Reported odds ratios [95%CI].

**p<0.01,

*p<0.05,

+ p<0.10.

Logistic multinomial regression models identified the associated factors with type of delivery, and more specifically, type of cesarean section received. (Table 4) The likelihood of planned cesarean deliveries is significantly associated with the education, Exp-pc, and indigenous household status. Having greater than or equal to 10 years of schooling, increases the likelihood of a cesarean section delivery (OR = 2.04; 95%CI: 1.30–3.20), in relation to women with no schooling, while belonging to fifth quintile of Exp-pc increases the probability of planned caesarean by 1.42, compared to women in the first quintile (95%CI: 1.11–1.83). Emergency cesarean sections, were statistically more likely in women who reported a health complication during pregnancy or child birth (OR = 1.32; 95%CI: 1.15–1.51 and OR: 6.93; 95%CI: 5.96, 8.05, respectively) and less likely among women with one, two or more children (OR: 0.58; 95%CI: 0.49–0.69 and OR: 0.34; 95%CI: 0.28–0.41, respectively), compared to nulliparous women. Finally, both planned and emergency cesarean deliveries are more likely in women aged 35 to 49 years than in women aged 12 to 19 years (OR: 3.26: 95%CI: 2.42–4.39, and OR: 2.13; 95%CI: 1.62–2.80, respectively) and in women who received care during delivery at a private institution, compared to those who received it at SS institutions (OR = 1.83; 95%CI: 1.35–2.47 and OR: 2.86; 95%CI: 2.12–3.87), for emergency and planned cesarean deliveries, respectively.

## Discussion

The present study analyzes the socio-demographic, prenatal care and clinical factors associated with the type delivery method a woman receives during her last pregnancy. The results confirmed a very high percentage of cesarean sections across all analyzed population groups, particularly when childbirth occurred in private sector facilities where 7 out of 10 pregnant women undergo a cesarean section. Additionally, the current rates of cesarean sections in public institutions surpass the 10–15% rate recommended by the WHO by three or four times and further surpass the 20% rate suggested by the Official Mexican Standard NOM-007-SSA2-2010 [32], [33].

Using an innovative approach with multinomial logistic regression models, the present study supports the existence of different socio-demographic and obstetric profiles among women who receive care for vaginal or cesarean delivery, confirming the suggestions made in previous studies [13], [34]. On one hand, the occurrence of emergency cesarean sections is elevated in women with pregnancy or delivery complications, as well as a history of abortion [34], while the occurrence is reduced in highly marginalized settings (defined in this study based on access to basic infrastructure services, housing conditions, education attainment, and wage earnings, at locality level) [13], [28]. On the other, planned cesarean deliveries are positively associated with years of schooling, a higher socioeconomic level, and age, elements which have been documented as being associated with a wider access to information about the pros and cons of this surgical procedure [35]–[37].

The relatively high percentage of emergency cesarean deliveries reported here (26.2%) contrasts with the figures reported in another study based on the WHO’s Global Maternal and Perinatal Health Survey, which suggest that in Latin American countries only 5% of the cesarean sections are emergencies, a discrepancy worth further discussion [15]. Interestingly, the findings of this study are very similar to the one reported by other national surveys, such as the 2009 National Survey on Demographic Dynamics (ENADID), which reported 22% of emergency cesarean deliveries [38]. This differences may be partly explained by the definition of “type of delivery” used in the surveys: while the 2012 ENSANUT and 2009 ENADID only considered two types of cesarean deliveries, the WHO survey included three types: elective, intra-delivery and emergency. Also, measurement based on self-report by the women in national surveys may lead to overestimation of the result [39], as it is difficult for the interviewees to adequately identify whether the procedure was planned or an emergency. There may not have been sufficient information transmitted to the women for her to understand the rationale at the decision point [38].

The findings of this study agree with those suggested in the international literature: the proportion of cesarean sections is significantly higher in the private sector than in public institutions [15], [40]. The study results suggest the potential role that market forces and economic incentives may be playing in the decision of carrying out the procedure or the existence of defensive medicine practices to avoid questioning regarding malpractices or lawsuits [41]–[44]. Previous studies, in the Latin American Region and in Mexico, have shown the relation between the increase of births through a caesarean and the expansion of private insurances of health that cover the costs of the cesarean section but not those of a vaginal childbirth, as well as the presence of diverse economic incentives related to private insurances [14], [45], [46], [47]. Although the percentage of the population with private insurance is low in Mexico (<1%), 21.5% of women analyzed received care from private providers during childbirth. However, further research is required in this area to identify the causes that prompt a delivery method choice.

Notably, the result suggests that women with higher adherence to prenatal care visits (≥4 ANC visits) have statistically higher probability of receiving institutional child birth care, using a cesarean section than women with less than 4 visits to a prenatal care provider. Nevertheless, this relation only was observed in the multinomial model for planned cesarean sections and needs to be more explored in future studies. In this sense, we would need to question the training and motivations of health care professionals in charge of providing delivery services within health institutions. Countries like the Netherlands and the United Kingdom, where care for a high percentage of child births is provided by midwives show a different reality in terms of the proportion of cesarean sections: 14.30% for the Netherlands, and 23.99% for the United Kingdom [48]. However, in order to achieve a significant presence of midwives in health institutions in Mexico, it is necessary to overcome certain previously documented challenges [49], [50], such as barriers on training and the development of these professionals and the factors that hinder their incorporation to the health system within a framework of reduced medicalization of delivery services. Reverting the growing trend of delivery by cesarean section across the country requires integrated strategies that include some of the following aspects, which were previously documented [51]:

1. To implement an effective monitoring system for obstetric practices to eliminate practices associated with unnecessary cesarean sections. Some potential measures are: an audit with feedback for the providers by their peers, reinforcement of the medical training, development of clinical practice guidelines, and a more sensitive quality indicator.
2. To extend the model of care for pregnant women to obstetric nurses and professional midwives [40]–[41].
3. To consolidate the regulation framework in the private delivery care market, directing it towards the elimination or reduction of the negative incentives for the unjustified increase of cesarean sections. Certain initiatives might include the use of the cesarean section rate as a criterion to adjust the codes, as well as specific premium payments to the public provider. Nevertheless, further research is required along, since experiences have been reported in other countries documenting the resistance by providers to the implementation of these initiatives [42].
4. To develop educational campaigns directed to the public in general, especially to reproductive aged women, to increase the information available regarding the advantages and disadvantages of the procedure [52].

The limitations of the study stem mainly from the availability of information and the cross-sectional nature of the survey used. Firstly, it was not possible to establish a direct connection between the decision of undergoing a cesarean section and the preceding obstetric conditions, a history of a previous cesarean delivery, or a history of infertility [16], [53]. Secondly, it was not possible to explore those elements of the offer of obstetric services associated to the increase in the cesarean delivery rates, or the preferences of women regarding the various delivery types, or the main causes that conditioned the choices made by the women. Thirdly, the potential overestimation of cesarean sections as a result of self-report regarding the type of care received at childbirth that has been discussed in previous studies [39]. Finally, information on women weight, height or body mass index at time of the last childbirth were not available, and the association between the higher body mass index and a higher cesarean section rate documented in previous studies [54], were not able to be explored in the analysis.

In conclusion, the findings of the present study support the existence of different socio-demographic and obstetric profiles among women who receive care for vaginal or cesarean delivery. The evidence presented here regarding the frequency and distribution of the institutional delivery care types in Mexico suggests the need to develop efficient strategies to reduce the number of cesarean sections- in both the public and the private sector- that are not clinically indicated, improving the overall quality of delivery care in the country. The consolidation of regulatory measures for the private sector cannot be delayed if providers are generating negative incentive structures which prompt the indiscriminate utilization of cesarean sections. Future studies must focus on attaining a deeper understanding of this phenomenon and on identifying the clinical and non-clinical factors that support the sustained and growing rates of pregnancies ending in cesarean sections.
